# Something about Engines and Hand-Pieces

**Published:** 1883-11

**Authors:** Norman W. Kingsley


### SOMETHING ABOUT ENGINES AND HAND-PIECES.

BY NORMAN W. KINGSLEY, D. D. S.

I desire to avail myself of the columns of the INDEPENDENT PRACTITIONER to make some comments upon the above subject.

It is becoming an important question as to how far machinery is a valuable adjunct in the practice of dentistry. If it be labor-saving, without a more than compensating loss of time or other objectionable feature, then it becomes positively an adjunct; but, if the most that can be said for it is that it furnishes but another method of doing the same thing, then its advantages are negative. There is a degree of fascination about machinery that encourages one to prefer its manipulation, rather than to accomplish the same object by more direct means.

If an artist discovered a method of painting pictures, or modeling statuary by machinery, equally well with former methods, he would no longer wait for inspiration, but would devote his brain power to directing the machine.

The status of machinery in dentistry is not fully settled; it is on trial; nevertheless certain things are definitely determined.

Machinery comes in competition with, and is intended to supersede hand labor. It will do this just so far as it will produce better results with less drain on brain or muscle power.

A machine that requires more time and care in its adjustment and management than it would take to do the same thing by hand, is not likely to become a labor-saving machine. One objection to any machine is that dependence upon it may entirely unfit one for doing certain things that should be performed directly by hand.

Attainable results being substantially equal among competing machines, that machine is the best which is made up of the fewest parts, has the least number of mechanical devices, and requires the least attention for keeping it in order. The value of the dental engine is a fixed and incontrovertible fact. Take the White dental engine for example; every part of it from pedal to cable is of the simplest character, while its possible movements are almost universal. It is a *labor-saving* machine in every sense of the word, requiring but little care to keep it in order, and producing better and quicker results than can be done by hand.

But the best engine is deprived of its value by an inferior hand-piece. Ten years of experience by thousands of dentists, has been quite enough to show that an imperfect hand-piece becomes one of the worst tools a dentist can attempt to use.

A score of different appliances have been put on the market and are undergoing trial upon their merits, and we can now, from experience, begin to form a correct judgment. The first made were slender and delicate in size, not much larger than a pocket pencil case. This was a compliment paid to dentists, who, like all skilled artisans, prefer delicate rather than clumsy tools.

But the necessity for superior instruments has shown that less than a certain limit in size will not give a reliable instrument. It is much the same with watches. The best possible time-keeper cannot be made in the smaller sizes. Experience has also shown that the larger hand pieces can be even more steadily manipulated and controlled than the smaller ones, and so any objection to the increase in size does not hold good. There are two kinds of hand-pieces in the market, which involve radically different principles. For the sake of distinction we will call them chucks, and latch pieces.

The principle of a chuck is so well known that a description is unnecessary. When properly applied it will hold whatever material it grasps, and revolve it around a center. A chuck, within certain limits, holds firmly and equally well whatever it incloses, irrespective of size. It is the simplest principle that has ever been devised for the purpose, and manifestly is the one principle which should be applied to the manufacture of hand-pieces. It is somewhat singular that any other principle should ever have been attempted. Of those now in the market made upon a different principle, that which is the latest and is intended to supersede all others is the " Cone journal hand-piece, No. 6," and I make this use of its name simply to illustrate my comments upon the principles involved in its construction.

I have never seen a machine intended for dentists, that was such a beautiful piece of mechanism, irrespective of its use. It is made up of nearly forty separate parts. Latches, locks, springs and slides appear to abound, and each and every part seems as delicately constructed and finely adjusted as the works of a watch.

This very abundance of parts and apparent complexity I regard as an objection. It is more liable to derangement, and more difficult to put in order and keep so. It is no small undertaking to separate all these pieces and put them together again correctly. It requires genius akin to that of the Yankee clock mender, and even if in every other respect it was a valuable tool, this one feature would deter many from its purchase. But the most serious objection to latch and lock hand-pieces is that heretofore every one of them began immediately to show such wear that the bur became unsteady, and in time completely valueless. I believe that a wear sufficient to produce unsteadiness is the inevitable concomitant of all hand pieces that are made with locks, latches, and springs. They may be made never so true in the start, but rust and wear begin immediately, at first imperceptibly, but gradually increasing until the instrument should be abandoned; in fact it should not be used one moment after unsteadiness in the bur can be detected. Another objection to the latch hand-piece lies in the necessity of having the bur stems or mandrels the same diameter, and slotted for fastening. The most minute variation in diameter gives unsteadiness. My own experience with hand pieces includes nearly every one which has been put upon the market, and out of all I have found but one which was not open to some, if not all of the above objections. A chuck hand-piece, when properly made, will obviate all such difficulties. It will run true for years, and within moderate limits will admit burs of different sizes equally well. I have been using one made by Hodge, for three years constantly, and it runs to-day as steadily and true as the day I bought it, and has not involved one penny of expense.

I do not say that no other hand-piece can equal it, but I do believe and say that it is impossible to equal it upon any other than the chuck principle, and I do not know of any other that is its equal.

